# The Needs of Patients With Chronic Disease in Transitional Care From Hospital to Home in Sudan: A Qualitative Study

**DOI:** 10.1177/11786329241249282

**Published:** 2024-05-14

**Authors:** Asma Mohamedsharif, Mayada Bastawi, Armin Gemperli

**Affiliations:** 1 Faculty of Health Sciences and Medicine, University of Lucerne, Lucerne, Switzerland; 2 University of Bahri, Khartoum, Sudan; 3 Center of Primary and Community Care, University of Lucerne, Lucerne, Switzerland

**Keywords:** Continuity of care, discharge management, low- and middle-income countries, transitional care, healthcare system

## Abstract

The growing burden of chronic non-communicable diseases demands improved post-discharge care. The Sudanese healthcare system faces challenges in providing coordinated care for patients with chronic conditions after hospital discharge. This qualitative study explored the experiences of patients with chronic disease in transitional care from hospital to home to identify improvement targets. Purposive sampling was used to interview 17 participants from different hospitals in Khartoum, Sudan. Audio recordings were transcribed and analyzed using principles of content analysis to identify themes and the relationship between them. Thematic analysis revealed 4 main themes describing the perceived needs of the patients. These were (1) feeling well-informed about post-discharge care goals and plans; (2) feeling cared for during hospital admission and follow-up visits; (3) feeling safe during the transitional care process; and (4) having access to follow-up services. This study highlights the importance of improving hospital patient education through effective communication to facilitate care transitions.

## Introduction

Chronic diseases are conditions, that are usually irreversible, long-lasting, and require lifelong care. Chronic diseases are mostly non-communicable diseases (NCDs) and include heart disease, diabetes, stroke, chronic lung disease, cancer, Alzheimer’s disease, and chronic kidney disease.^1^ Since 1990, there has been a marked shift toward a greater proportion of the global burden of disease attributable to non-communicable diseases and injuries.^2^ In 11 countries more than half of the disease burden was from years of healthy life lost due to disability (YLDs) of non-communicable diseases and injuries in 2019.^2^ Annually, 17 million people die from an NCD before reaching the age of 70; 86% of these premature deaths occur in low- and middle-income countries.^3^ Chronic non-communicable diseases are also a significant challenge for the healthcare system in Sudan.^4^ Patients with chronic diseases are at risk when they are transferred between different healthcare system settings. Discharging patients from hospitals poses a threat to their safety, as it may increase the chance of complications and readmissions and requires a high level of coordination.^5^

Research indicates that a significant number of patients, ranging from one-quarter to one-third, are rehospitalized due to preventable complications.^6^ Approximately 1 in 5 patients encounter adverse events after hospital discharge, which can lead to unplanned readmissions, and, in severe cases, death.^6-8^ The median hospital 30-day risk-standardized readmission rate is one of the essential measures of transition of care quality.^9^ Unplanned readmission signals a failure in the hospital discharge process, the patient’s incapability for self-care, the insufficient quality of care in the next community setting (such as physician practices, home healthcare, or skilled nursing facilities), or the absence of appropriate care resources for high-risk patients.^10,11^ Hospital readmissions cost an approximate annual figure of $26 billion in the US.^12^ A “transitional care strategy” refers to a set of interventions implemented before hospital discharge to ensure that patients receive timely, safe, and uninterrupted healthcare services during their transitions from one care setting to another, such as from the hospital to home.^13^ Improving post-discharge transitional care can enhance patient outcomes, reduce mortality, and lower healthcare costs.^14,15^ It is essential to tailor interventions that aim to improve post-discharge transitional care to the unique context of each health system and the specific values, preferences, and needs of the target populations. This patient-centered approach would ensure that the interventions are more effective and better suited to the challenges individuals face during the critical period following hospital admission.^16^ Understanding patient-defined needs and concerns is crucial for the design and evaluation of innovations.^17,18^

Improving transitional care quality necessitates an in-depth understanding of patient experiences and perceptions to recognize areas of intervention enhancement. This will lead to greater care adherence in the long run.^19,20^ Studies show that multiple factors, including social and ecological conditions and societal upheavals, contributed to the sustainability of interventions in sub-Saharan Africa.^21^ Although there has been an increase in qualitative research examining the challenges faced by patients with chronic diseases and shortcomings in transitional care,^17,22-24^ there is still a significant knowledge gap when it comes to understanding the post-discharge experiences of patients in low-income countries. Effective transitional care strategies must take into account differences in healthcare resources and cultural factors while balancing local policy development and standardization.^25,26^ In fragile health systems, care for patients with chronic disease tends to be uncoordinated and fragmented.^27^ In Sudan, the current process for transitional care from hospital to home typically includes verbal health education before discharge and a follow-up appointment within 2 weeks at the hospital outpatient clinic. The referral system is weak as hospitals accept patients without being referred from lower-level facilities such as primary healthcare centers (PHC). Usually, patients are not referred to PHC after being discharged from the hospital. The follow-up care is provided in the outpatient clinic by the same team that was responsible for inpatient care.^28^ In Sudan, the perceptions of patients with chronic diseases about transitional care from hospital to home have received little attention. In our preliminary literature search, we found few studies that focus on satisfaction rating scores of the quality of services without exploring underlying needs during the specific period after discharge.^29,30^

This study aims to explore patients with chronic disease needs in transitional care from hospital to home in Sudan. Identifying these needs will inform interventions aimed at improving transitional care and enhancing continuity of care for Sudanese patients and potentially other low-income settings worldwide.

## Methods

### Study design

This qualitative study used semi-structured interviews to explore the needs of patients with chronic diseases in transitional care from hospital to home in Sudan. The reporting follows the Consolidated Criteria for Reporting Qualitative Research (COREQ) checklist for qualitative studies^31^ (Supplement S1).

### Setting and participants

The Sudanese health care system is a federal system, the health and other sectors manage service delivery at the federal, state, and local levels. Each state has its own State Ministry of Health in the health sector. The Federal Ministry of Health is responsible for setting national legislation, policies, priorities, standards, and training healthcare workers. It has an overall stewardship function over the state ministries of health. The Federal Ministry of Health is responsible for declaring and controlling epidemics. States have some flexibility and autonomy to develop their own health plans and legislation, but they do so in close coordination with the Federal Ministry of Health. States are responsible for the performance of the localities in their state. Localities are responsible for delivering primary health care services and aggregating health services by facilities in their districts.^32^

**Figure 1.**
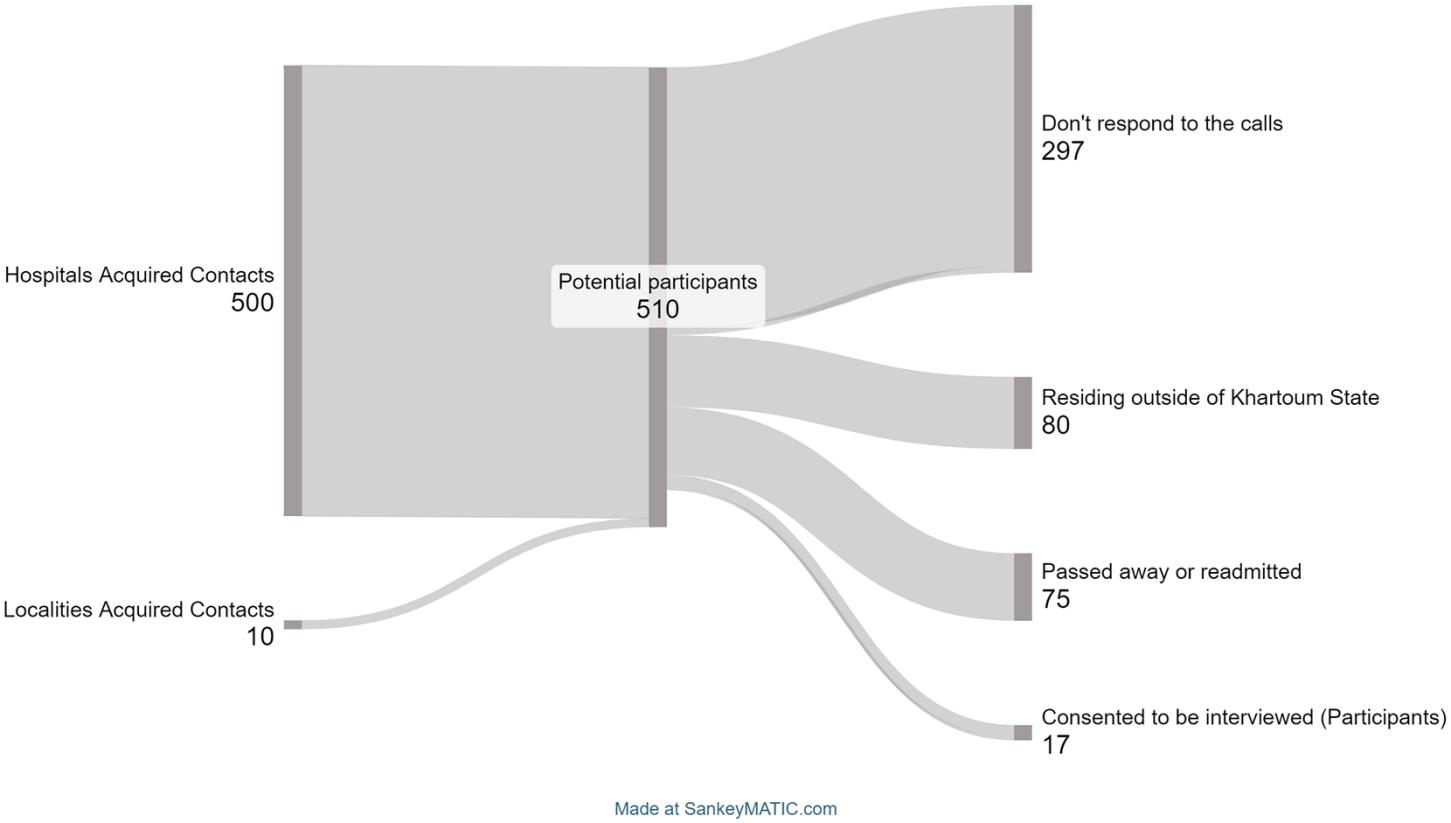
Participants recruitment process.

This study included participants who were patients with a chronic disease discharged from hospitals in Khartoum State. The inclusion criteria were as follows: participants had to be aged ⩾18 years, coping with at least 1 chronic disease (e.g. diabetes, coronary heart disease, chronic obstructive pulmonary disease, stroke, cancer), have experienced at least 1 transfer from hospital to home within the past 2 weeks to 3 months, and be able to communicate verbally (i.e., no language impairment or aphasia) in Sudanese Arabic language. We excluded participants with mental capacity issues or who lived outside of Khartoum State. To recruit participants who had been discharged from the hospital, and given the lack of an NCD registry, we used non-probability sampling – a purposive sampling method. We included patients discharged from 3 major hospitals, Bahri Hospital (a general public hospital), Ibrahim Malik Hospital (a general public hospital), and Ahmed Qasim Cardiac Center (a specialized public hospital). These hospitals cover the healthcare needs in the main districts in Khartoum State. As shown in Figure 1, we acquired contact details of nearly 500 patients who met the inclusion criteria, including data on their reason for hospital admission, residential address, and telephone number from the hospital registry office. Amongst them, 290 contact were not responding to the calls or had misspelled phone numbers. In addition, 80 patients were residing outside of Khartoum State, and 75 patients have either passed away, been readmitted, or no longer met the inclusion criteria after a re-examination of the documentation. A total of 14 patients consented to be interviewed. To further expand the participant pool, we contacted the local residential associates, namely the discrete associates of Burri and Bahri Alshaabiya. They offered the contact details of patients who had been admitted to hospitals and met our inclusion criteria. Through their assistance, we identified 10 patients who were admitted to one of the following hospitals in Khartoum State: Almualim Hospital, Sudan Heart Center, or Royal Care Internal Hospital. Seven patients did not respond to the call, while 3 accepted our invitation to participate. All those who met the inclusion criteria were contacted by telephone to explain the objectives of the study and to request their participation. Those who were subsequently interviewed were again informed about the study goals and assured that all data would be treated in strict confidence. The literature in this field has indicated that saturation is typically achieved between 12 and 20 participants.^33^ We conducted interviews with 17 participants.

### Data collection and analysis

The second author (M.B.) carried out semi-structured interviews, which were face-to-face interviews, in the homes of the participants in the presence of their families.

The study period was 3 months, dedicated to participant recruitment, and conducting interviews. The interviewswere conducted in Sudanese Arabic language and took place in March 2023, each interview lasting between 30 and 35 minutes. An interview guide was developed based on the literature on transitional care (Supplement S2). Participants were asked to reflect on their discharge experience, with a particular emphasis on the following topics: receiving and understanding information for self-management, relationship with the healthcare providers, follow-up visit after discharge, family involvement, and perception of the healthcare facility. The interview guide underwent a single pilot interview to ensure efficacy. During our pilot, it became evident that participants were hesitant to express their views openly. To address this, we adapted the questioning approach by broadening and shortening certain questions to reduce directness to their experiences.

All interviews were recorded and transcribed verbatim. We used investigators’ triangulation, and the most important statements were independently coded by 2 analysts (A.M. and M.B.). The data were analyzed using qualitative content analysis according to Graneheim and Lundman.^34^ In this study, we employed qualitative content analysis as a systematic and objective method to extract insights from written data. The analysis included both manifest and latent content examination. The manifest analysis focused on identifying explicit elements, while latent analysis involved interpreting underlying meanings. We aimed to acquire a comprehensive understanding and knowledge of the subject being investigated. This approach proved valuable when exploring conservative viewpoints, allowing us to uncover participants’ unspoken thoughts and beliefs.^34^ Disagreements between the 2 analysts about the underlying meaning of the defied code were resolved through discussion meetings. The analysis consisted of 3 steps: content line-by-line coding, development of categories from group codes, and development of the analytical themes.^35^ Thematic analysis was used as a qualitative method to identify, analyze, and interprete patterns of meaning within the identified categories and codes.^36^ We used the ATLAS.ti application, version 2023, for coding and analysis.^37^ Sudanese Arabic quotations were translated into English for publication while preserving the style and meaning of the quote. In the patient’s narratives, a need was acknowledged if it was either fulfilled or unfulfilled in their experience. When a patient identifies a particular need, it indicates its value and the quality of care from the patient’s point of view. For instance, 1 patient mentioned that the renal unit made exceptions for them, recognizing their distinct and significant case. However, this same point was also mentioned as a drawback of the service, as another patient expressed dissatisfaction with not feeling treated differently despite their unique circumstances, which they believed warranted exceptional attention M.B. did not have a relationship with the participants before the study commencement. The analysis consisted of 3 steps: content line-by-line coding, development of categories from group codes, and development of the analytical themes (thematic analysis).

### Ethical aspects

All participants provided written informed consent to participate in the study. The National Health Research Ethics Review Committee approved the study No. (6-11-22). The principles of good practice and the provisions of the Declaration of Helsinki and its subsequent revisions were upheld throughout the study. The data were treated confidentially and anonymized for analysis.

## Results

**Figure 2.**
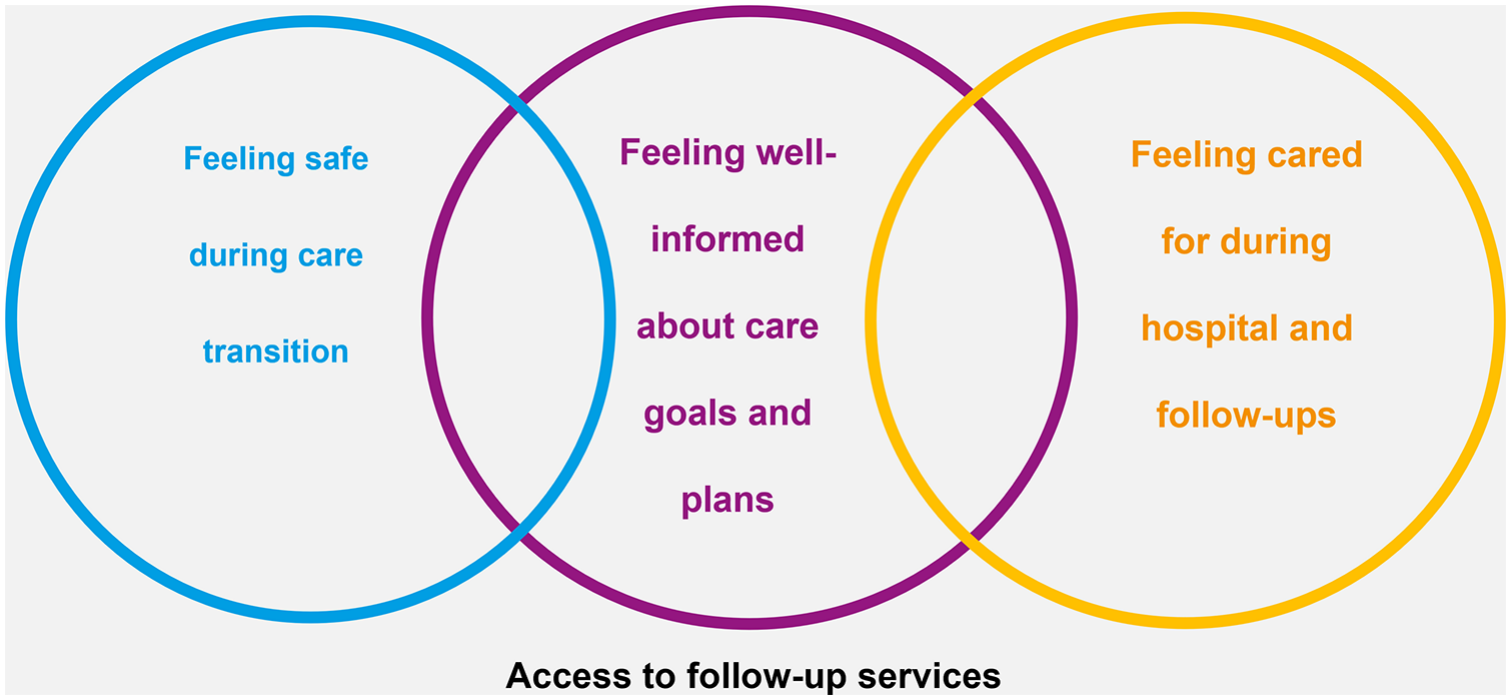
Representation of the four most important transitional care needs perceived by patients and how they might be interrelated.

The study participants were between 23 and 70 years of age and included 11 females and 6 males, predominantly married (13 out of 17). The chronic conditions identified encompass diabetes mellitus type 2 (7 participants), chronic kidney disease (2 participants), hypertension (3 participants), congestive heart failure (3 participants), and ischemic heart disease (2 participants), renal cell carcinoma (1 participant), among other conditions. Employment statuses varied from housewives (6 participants) and students (2 participants) to other occupations, with educational achievements ranging from none (5 participants) to post-university degrees (1 participant). Financial support was mainly families (16 participants), with some relying on themselves (5 participants). Healthcare was sought across different facilities: general public hospitals (8 participants), private hospitals (4 participants), specialized public hospitals (4 participants), and specialized military hospital (1 participant). Details of the participant characteristics are presented in Supplement S3. The thematic analysis revealed 4 major themes that describe the patients’ perceived transitional care needs. Table 1 provides an overview of the 36 subcategories, 13 main categories, and the overall 4 themes (Figure 2).

**Table 1.** Overview of subcategories, categories, and overall themes.

Theme	Categories	Subcategories
Feeling well-informed about post-discharge care goals and plans	Adequacy of information	Adequate information provision
		Provision of extra information
		Linking disease and social life
	Family empowerment	Role of family in patient care
		Perception of family’s level of information
	Implications of feeling well-informed	Decision on the follow-up visit
		Satisfaction with quality of care
		Feel cared for and safe
Feeling cared for during hospital admission and follow-up visits	Desire to feel cared for and valued	Genuine interest and concern
		Personalized care
		Feeling valued as an individual
	Attitude of healthcare providers	Warmth and friendliness
		Respect and dignity
		Healthcare providers’ mental well-being
		Attentive behavior
		Reassurance
	Implications of feeling cared for	Health improvements
		Satisfaction with quality of care
Feeling safe during the transitional care process	Appraisal of care	Realistic expectations
		Safety concerns
		Reputation of doctors and hospital
	Trust	Building trust during hospital admission
		Doctors’ responsiveness and proactivity
		Influence of non-medical aspects on perception of care
		Close medical monitoring
		Consideration of cultural, religious, and social needs
	Implications of feeling safe	Seeking care from the same healthcare provider
		Seeking validation from other healthcare providers
		Traditional medicine preference
Access to follow-up services	Affordability	Affordability of services
		Seeking care from the private sector
	Availability	Availability of essential services
	Accessibility	Accessibility of physical environment
		Distance to follow-up facilities
	Accommodation	Cleanliness of physical environment
		Waiting time
		Crowdedness
	Acceptability	Equitable treatment
		Suitability of primary healthcare

### Feeling well-informed about post-discharge goals and plans

#### Adequacy of information

A notable deficit was reported in information provision, particularly about long-term follow-up beyond the first 2 weeks after discharge. Also, there was insufficient information about self-management and the fear of complications. Some participants were not informed about the side effects of their medications, the warning signs, and the prognosis, and some were not even informed about the diagnosis. The lack of satisfactory quality and quantity of information leads the participants to seek additional information themselves and to fill the gap with sources such as the internet, peers, or family. Educational level and health literacy may influence the choice of information sources.

**Table:** 

“He didn’t tell me anything. No, they didn’t say anything to me, nothing about diagnosis nor follow-up plan, nothing”	P3
“I had to go to the internet and read about this issue, and everything, I could be simply informed by the doctor”	P15
“Oh GOD, the issue started with me; it was severe abdominal pain that kept coming. At first, it used to come like once every year, then it became every 6 months. After a while, it started happening every 2 months. You know, when my mother passed away, it became a daily thing, morning and evening. Then, everything nothing I ate led to pain; nothing worked for me. Even milk, which should be easy on the stomach, and yogurt caused pain”	P11

#### Family empowerment

Family members play a vital role in the care of patients, both during hospital admission when they assist in bringing medications from the pharmacy and transferring lab samples, and as informal caregivers after hospital discharge. The participants have a deep appreciation for the role of their family members during and after hospital discharge. However, the participants often perceived that their families were not adequately informed. There is a lack of intentional involvement by healthcare providers to empower both the participants and their families with relevant information about the transition from hospital to home.

**Table:** 

“Oh my goodness, my family was part of everything! I was nothing but a bedridden patient, and they were doing everything, they were (mumbling) getting medications from the pharmacy or taking samples to the lab”	P9
“They didn’t explain to my caregivers anything, maybe they wrote things in the medical record, but we don’t know”	P2

#### Implications of feeling well-informed

The participants and their families value being knowledgeable about their health, starting from the diagnosis to understanding the causes of their condition and factors that can worsen it. The participants wished to be informed about the goals of the follow-up visits and the care plans after hospital discharge. The participants expressed dissatisfaction with not being well-informed, arguing that they need to understand the purpose of doctors’ instructions about the follow-up visits before deciding to attend or not. Feeling well-informed has implications for other healthcare needs: It builds trust in healthcare providers and creates a sense of being cared for.

**Table:** 

“God, if only they would have explained it to us (patients). When the doctor explains it to you, then you would make efforts to get him (next time)”	P9
“The doctors themselves, they showed me and explained it to me. I mean, the (mumbling) specialists I used to visit, they told me, “For example, they said because you have this, we might have to do this exploratory catheterization procedure, and we’ll see what’s going on with you.” They made it clear to me, and they explained that the catheterization is like this and this and this and this.	P11
From the perspective of direct care I have received, if I ever said something I didn’t like, I’d be lying to you because I understand everything, you know. Even when the doctor comes to attend to you, they’ll show you this . . .. . .. . ... Perfection belongs to God, they were really good (mumbles)"	
“I mean whenever the patient knows what they have, the doctor makes it clear to them. The person should be aware, and the doctor should show it to them honestly, and they have it available. The doctor is reassured, meaning that they know that what they do even (stuttering) is also something important. I mean, I didn’t need to ask whether it’s like this or that, but this is what I imagine is the most important thing for the doctor to. . . (stutter). . . show it to the patient”

### Feeling cared for during the hospital admission and the follow-up visit

#### Desire to feel cared for and valued

A dominant theme among the participants was the desire to feel cared for and valued in their interactions with healthcare providers. The meaning of feeling cared for goes beyond receiving prescribed medication. The participants emphasized the importance of experiencing genuine care through personalized interactions with healthcare providers that demonstrate a relationship and understanding of their unique circumstances. Feeling important and valued as an individual had a significant impact on their perception of the quality of follow-up care they received and reduced loss to follow-up care.

**Table:** 

“What do I want in care? The feeling that there is care here”	P15
“They didn’t care at all. The first thing, when you arrive, they see you just like anyone else, like a normal patient. They don’t care that this person has been through something catastrophic. They don’t say we should stand by him so that he can get reassurance. Nothing, they consider you like any other patient who came to the clinic”	P8
“You feel like you’re an important person, and someone comes asking about (stuttering) this is self-like treatment”	P4

#### Attitude of healthcare providers

The participants valued healthcare providers who demonstrated a genuine interest in their health by asking about their well-being and addressing their concerns. The reassurance provided by healthcare professionals played an important role in fostering a sense of comfort and confidence in follow-up care. The participants found comfort in the gentle treatment, the simple gestures of a warm and welcoming facial expression, and the friendly manner of the healthcare team. Attentive behavior, such as actively listening to their questions and responding to their concerns, was highlighted as an essential component of feeling cared for. Patients identified respect and dignity as critical aspects of their care experience and relevant to adhering to follow-up care.

**Table:** 

“I mean he meets people with warm greetings”	P3
“Some doctors don’t accept you are coming to measure your blood pressure. They (hesitated), and the doctor himself (hesitated) when I came to measure twice or three times. He said, ‘You should measure from month to month; what brings you daily? ’ ”	P8
“The doctors themselves were nervous and anxious, and (hesitated), and there were a lot of people and noise. Anyway, I stood up and said to her, ‘Look, I . . .’ ”	P9
“When I visit a doctor and the doctor doesn’t give me proper attention and appears distracted, (frustrated low voice pitch), he does not tell you what is wrong with you, just he said the blood pressure is high. . .no further explanation”	P9
“In the morning, indeed, and they wanted to check on me, their treatment was very kind toward me, like, ‘Don’t worry at all, there’s nothing to be concerned about’ ”	P12

#### Implications of feeling cared for

Despite certain medical limitations that prevented definitive treatment in some healthcare facilities, the participants still expressed deep appreciation for the caring behavior and welcoming attitude displayed by healthcare providers during their hospital admission and beyond. The participants directly linked the gentle and empathetic behaviors of their healthcare providers to a positive moral impact on their health improvement and overall well-being.

**Table:** 

“When the doctor comes to you smiling and using sweet words to attract the patient, and he says, ‘What’s going on with you, my dear?’ and asks about your condition, believe me, I feel like as soon as I saw this personality, my pain disappeared”	P12
“No, I (hesitated) and I had already gone to the (hospital name),. . .. . .. . .. . ., and I swear it has excellent care. I mean, the care I found there, I was really pleased with this treatment”	P11

### Feeling safe during the transitional care process

#### Appraisal of care

The participant’s main expectation when using healthcare services is to receive professional and effective treatment for their illnesses. The reputation of both the healthcare provider and the hospital plays a vital role in ensuring that patients have confidence that they will receive quality medical care from competent physicians. Similarly, for follow-up care, patients look for competent healthcare providers whom they can trust to provide the best possible care.

**Table:** 

“I would like the doctor to provide me with easy and effective treatment”	P14
“It was a problem; I mean, the doctors themselves, some of them are not specialists. It’s all (unclear pitch voice), they dealt with the situation as it was. They try to send you to (unclear), to Khartoum (big hospital)”	P10
“Um, um, and they have good specialists. In the past, we used to go to (unclear) in the big hospital in Khartoum for (unclear)”	P10

#### Trust

Patients’ experiences with healthcare services, particularly during their hospital admission, have a significant impact on their confidence in the healthcare system, and subsequently, on their willingness to seek follow-up care. Trust in healthcare providers during hospital admission is crucial for the participants and their families. They value the responsiveness and proactivity of healthcare providers during their stay and appreciate close medical monitoring. They also valued the timely administration of medication during hospital admission. It is also important that their cultural, religious, and social values are considered. Even seemingly unrelated aspects, such as the quality of the food provided during their hospital admission, can influence their perception of the overall medical care they receive, and subsequently their adherence to follow-up care.

**Table:** 

“The monitoring care is a thing in the hospital, what we have in terms of monitoring care, there’s nothing. We need to do the monitoring of the infusion pump by ourselves (the patient and his caregiver) I mean, there’s no real attention to the issue itself”	P15
“People from nutrition come and ask you, ‘Does this suit you? What do you want in terms of salt and sweet?’ I mean, was there care”	P11
“This care, firstly, you’re lying down. It’s all about a half-hour. These people (unclear) in . . .(hospital name), they come to you from morning until evening, and from evening until morning, like that. If they come in the morning, they’ll come again in the evening. And then in the evening, around this time, they’ll come again”	P8
“I was looking for the ritual stones for ablution before prayer, but, Mashallah, it is also available, and they brought the stones from a small cabinet. . . There’s nothing else better”	P11

#### Implications of feeling safe

If the participants feel safe when they are admitted to the hospital, there will be short- and long-term benefits. In the short term, the participants may prefer to be seen at follow-up visits by the same healthcare provider who initially treated them during hospital admission. They may seek their diagnosis or treatment plan from alternative providers. In the long term, a lack of trust may lead the participants to turn to traditional medicine as their first point of care or to avoid healthcare services altogether.

**Table:** 

“they took me to (. . ...) the clinic, but it wasn’t the same doctor.’ I said, ‘Look, I’m under the care of a specific doctor who knows what’s going on. I have the latest tests and such.’” I refused because my doctor who had my medical records,. . .. . . Okay, I got annoyed with the care in the clinic. I got frustrated. So, I just continued with the same medication”	P6
“They wanted me (her sister) to go to Egypt. They told me it was possible (the diagnosis). I found the people in Egypt saying the same things they told me in Sudan”	P1
“I swear, it’s in my nature. God created me this way. I don’t like all the (stutter), I don’t mean to say I don’t like them. I always seek treatment through natural remedies, the local ones, you know, someone with a stomach ache, I ask for ‘halabah,’ someone with a specific issue, I seek help from them. If someone has a problem, they give me a loan. I’ve always seen these herbal remedies, I love natural things”	P12

### Access to follow-up services

#### Affordability

Affordability of services emerged as a critical need when patients were considering follow-up care. Although the cost of follow-up care services was a concern for participants, they were willing to use the more expensive private sector to ensure quality care and respectful and attentive behavior by healthcare providers.

**Table:** 

“Just two options. If you have insurance, they’ll write a receipt for you. If you don’t have insurance, they ask you to bring money”	P2
“The doctors in the private clinic asked me to save my money, and he said to me, you can get the same service for free. I told him if the free government service treated us well, would have gone to private? But when we go to free service, he treated us badly, the medicine they provide instead of reducing our problems, he increases our blood pressure”	P8

#### Availability

The participants emphasized the importance of the availability of essential follow-up services, including lab tests and essential medications. They pointed out that PHC facilities are not well-equipped, and that unstable electrical power supply dramatically affects the availability of laboratory tests.

**Table:** 

“I hope that everything is available there (PHC). Sometimes the electricity goes out, sometimes there’s no water, you know”	P11
“Everything should be available there (PHC). Sometimes they don’t have things like diabetes medicines, or they might not even have medication for high hypertension. In such cases, you have to go to the center”	P1

#### Accessibility

A barrier to care continuation was the poor physical accessibility, with a lack of wheelchair access or uncomfortable seating. The distance to follow-up facilities was a crucial factor in determining whether patients would continue follow-up care or not.

**Table:** 

“Yes, it is a good hospital. I can’t walk you need to use transportation, three changes”	P7

#### Accommodation

The healthcare facility’s physical environment, with unclean areas or bathrooms, poor infrastructure, or unstable electricity affects participants’ decision to continue care. Patients expressed concern that long waiting times for follow-up visits and overcrowding could have a negative impact on their health.

**Table:** 

“The hospital was dirty and stuffy, I mean, the environment wasn’t good, but. . .”	P4
“Last time I went to the clinic I went at 7 am, we came back around this time (6 pm)”	P9
“It’s suffering, I mean, as much as I go to them, by God, it’s a struggle. It’s a struggle, meaning there are a lot of people”	P1

#### Acceptability

Participants stressed the importance of inequitable treatment based on financial status when seeking follow-up care. They perceived PHC as an appropriate facility for managing simple illnesses and injuries.

**Table:** 

“This is supposed to be a humanitarian act. Doctors are supposed to care for the patient equally, not based on their money or their appearance or. . .”	P8
“The health center was only suitable for changing for small wounds”	P2

## Discussion

The purpose of the study was to explore the needs of patients with chronic disease in transitional care from hospital to home care. Thematic analysis revealed 4 major themes that describe the patients’ perceived needs. The identified themes were feeling well-informed about post-discharge care goals and plans, feeling cared for during hospital admission and follow-up visits, feeling safe during the transitional care process, and having access to follow-up services.

In Sudan, patients were usually advised to return to the hospital for outpatient follow-up visits. However, patients often lack information about the long-term plan, resulting in low adherence.^38^ Studies have shown that poorly informed patients with chronic diseases show little responsibility for decisions about their health and have poor adherence to self-management.^20^ Patients eagerly try to fill the gap with any information based on their capacity to understand, which may not be accurate. For patients with low levels of education and health literacy, healthcare providers may need to make a greater effort to simplify patient information, which requires additional time. Staff shortage and overcrowding make this more challenging. Literature shows that Sudan’s human resources for health are inadequate, poorly distributed, underfunded, and weakly managed. The physician-to-population ratio stands at 1:10,000.^39^ In this study, we found that feeling informed builds trust in healthcare providers and gives a sense of being cared for. The participants tend to associate their illness or new symptoms with social events in their lives. Healthcare providers, on the other hand, tend to stick to biomedical explanations of the disease. The mismatch between perceived social causes and medical explanations creates dissatisfaction and contributes to the feeling of not being well-informed. This underscores the importance of adopting a patient-centered approach to patient care. Engel^40^ introduced the biopsychosocial model (BPS) as an alternative to replacing the biomedical model. By combining qualitative factors such as thoughts, beliefs, behaviors, social context, and interactions with biological processes, disease, and disability can be better understood and managed by patients.^41^

The families of the participants play an important role in patient care. On admission, they transfer laboratory samples or buy medicines from the pharmacy. The high level of involvement of family members in day-to-day patient care requires them to be well-informed. Patients appreciate the social and family support they receive during their hospital stay and throughout the course of their illness. Well-informed family members are an opportunity to improve patient self-management.

Feeling cared for is a universal patient need. Studies have shown that patients perceive feeling cared for by healthcare providers as an essential outcome for safe transitions of care.^42,43^ A discrete choice experiment on preferences for care delivery among 3003 Tanzanian women found that the strongest predictor of health facility preference was being treated kindly by healthcare providers, followed by having a healthcare provider with excellent medical knowledge and modern medical equipment and drugs.^44^ Feeling cared for means that the healthcare providers show patients that they care through their daily communication. Reassurance, inquiring about their well-being, facial expressions, attentiveness, and respect show patients how healthcare providers behave. Another discrete choice experiment among HIV patients in Zambia who were lost to follow-up care found that patients were willing to wait 19 hours longer or travel 45 km further to see nice rather than rude providers.^45^

An underlying, central theme that emerged in this study, was the feeling of safety during transitional care. The trust patients have in their healthcare providers is associated with patient satisfaction, health behavior, quality of life, and symptom severity.^46-48^ Participants expressed a preference for being treated by the same healthcare providers once they have developed trust and feel safe.^49^ Trust can be transferred to other healthcare providers, using communication tools.^50^ In Sudan, transferability of trust is limited due to underdeveloped communication tools among healthcare providers, even of providers within the same facility.^4^ When developing planning and improvement strategies for transitional care, it is important to acknowledge that healthcare trust is influenced by both family and the community.^51^

Feeling cared for and feeling safe among participants are similarly described as needs in the literature.^42-46,48,49,51^ This highlights a shared foundation of basic needs. However, how patients expect these needs to be met may vary due to cultural differences, religion, and social values. For instance, many studies identified the need for feeling safe with service providers as a basic need among participants. However, how individuals cope in the absence of these needs can vary. In our study, one of the participants mentioned that they turn to herbal remedies and alternative medicine, which may not be the strategy in other cultures. Furthermore, how the participants perceived the feeling of being cared for was influenced by the consideration of their religious values. For instance, a patient praised a hospital for having the stone for Tayammum (dry ablution) for prayer. This highlights the importance of considering cultural and religious needs when providing services and recommendations.

The participants perceived PHC as suitable for handling uncomplicated illnesses and injuries. Currently, the norm in Sudan is for patients to return to hospitals for follow-up care, although this could be effectively managed at the PHC level. In a study conducted in one of the states in Sudan, quality of care and ratings of the general practitioner’s care were found to be significant determinants for seeking outside the referral network.^52^ Maximizing the effectiveness of PHC requires significant changes in approach. The ratio of PHC facilities to population varies from 1:3,000 people in the Northern State to 1:21,000 people in South Darfur, compared to the planned 1:5,000 population. According to the Sudan PHC Mapping Survey,^53^ 24% of PHC facilities provide the PHC minimum package, while only 9% provide the comprehensive PHC package. There is an urgent need to improve the quality of PHC services, in addition to integrating the Package of Essential Noncommunicable Disease (PEN) interventions into PHC frameworks.^54^ This study has shown that participants, regardless of their financial status, are willing to pay more out-of-pocket for private healthcare services to receive the specific care attributes that meet their preferences and needs. In a discrete choice experiment conducted on persons with hypertension and diabetes in rural Uganda, respondents were willing to pay more to attend facilities that offered peer-support groups, friendly healthcare providers with low staff turnover, and greater availability of medications.^55^

### Study implication and future directions

The practical implication of this study could be an intervention aimed at enhancing patient education protocols during hospital admission.^56^ Implementing effective strategies for doctor-patient communication and adhering to best practices can build a foundation of trust between patients and their healthcare providers. Patients need to be involved in the future planning and co-design of healthcare services.^18^ Future studies should focus on the development of a robust tool to measure the quality of health services specifically tailored to the needs of patients with chronic diseases, using the identified attributes as key metrics. Additionally, we recommend a discrete choice experiment to quantitatively assess the relative importance of the different needs, as identified in this research, in influencing patients’ decisions about continuing care after hospital discharge. A discrete choice experiment can quantify participants’ preferences for different types of healthcare financing to meet these needs.

### Study strengths and limitations

A strength of our study was that it included a heterogeneous sample of participants with different ages and types of chronic diseases. The study had a diverse representation from different hospitals in Khartoum State. The pilot test made us aware that the participants would be rather conservative in their perceptions of the healthcare service. We asked the participants for the most convenient setting for the interview, we prepared questions to ask from different angles, and in the analysis, we used both manifest and latent content analysis.^34^ The credibility of the study was increased by triangulation, where agreement between the 2 analysts was sought. A limitation is the study’s self-selection bias of patients who agreed to participate. Those who did not wish to participate, who were not approachable, or who resided outside Khartoum State may have had different healthcare experiences than those we described.

## Conclusions

This study highlights the importance of comprehensive information about care goals and plans after discharge, the importance of feeling genuinely cared for both during hospital admission and at follow-up visits, the need for a sense of safety during the transitional care process and improving access to follow-up services. Central to these themes is the crucial role of effective communication, particularly in the early stages of hospital admission, as it lays the foundation for trust between patients and healthcare providers. Future research could focus on generalizing these findings using quantitative measures and developing a comprehensive tool that incorporates these needs to measure the quality of care for patients with chronic diseases.

## Supplemental Material

sj-docx-1-his-10.1177_11786329241249282 – Supplemental material for The Needs of Patients With Chronic Disease in Transitional Care From Hospital to Home in Sudan: A Qualitative Study Supplemental material, sj-docx-1-his-10.1177_11786329241249282 for The Needs of Patients With Chronic Disease in Transitional Care From Hospital to Home in Sudan: A Qualitative Study by Asma Mohamedsharif, Mayada Bastawi and Armin Gemperli in Health Services Insights

sj-docx-2-his-10.1177_11786329241249282 – Supplemental material for The Needs of Patients With Chronic Disease in Transitional Care From Hospital to Home in Sudan: A Qualitative Study Supplemental material, sj-docx-2-his-10.1177_11786329241249282 for The Needs of Patients With Chronic Disease in Transitional Care From Hospital to Home in Sudan: A Qualitative Study by Asma Mohamedsharif, Mayada Bastawi and Armin Gemperli in Health Services Insights

sj-docx-3-his-10.1177_11786329241249282 – Supplemental material for The Needs of Patients With Chronic Disease in Transitional Care From Hospital to Home in Sudan: A Qualitative Study Supplemental material, sj-docx-3-his-10.1177_11786329241249282 for The Needs of Patients With Chronic Disease in Transitional Care From Hospital to Home in Sudan: A Qualitative Study by Asma Mohamedsharif, Mayada Bastawi and Armin Gemperli in Health Services Insights
