# Supplementary material for: The Needs of Patients With Chronic Disease in Transitional Care From Hospital to Home in Sudan: A Qualitative Study
Source: Health Serv Insights. 2024 May 14;17:11786329241249282. doi: 10.1177/11786329241249282 (PMC11092543; doi:10.1177/11786329241249282)
Supplement: sj-docx-2-his-10.1177_11786329241249282 – Supplemental material for The Needs of Patients With Chronic Disease in Transitional Care From Hospital to Home in Sudan: A Qualitative Study [file sj-docx-2-his-10.1177_11786329241249282.docx]

# Short interview guide:

| **Intro:** |
| --- |
| **Receive and understand the information:**   - Information regards: symptoms to look out for at home etc - Received vs wanted |
| **Self-management:**   - Future tests/monitoring of the condition. - Medications management. |
| **Caregiver involvement:**  How the caregiver was involved in discharge and after discharge? |
| **Continuity of care and follow-up care:**   - Scheduled appointment - Transportation - **Cost** |
| **Experience of the patient with the healthcare providers:**   - Experience: Like and dislike - Expectations from healthcare provider |
| **Relationship with health service:**   - Experience: Like and dislike - Expectations from health service primary health service. |
| **Cost of treatment:**   - Health insurance - Out of pocket |
| **Final:**   - What make this better experience? |

# Detailed interview guide:

| Narrative of the experience | I want to introduce myself; I am …………………….  I am interested in learning more about the life stories of people who has been admitted to the hospital and have chronic disease  I would like to listen to *your* story, I want to know more about your experiences in hospital, at the time of the discharge and after discharge home.  I am interested in your needs, your support over time, experiences and events that were particularly meaningful for you and your family.  While I have some questions to guide us along in the interview process, I am interested in hearing about anything else that *you* think would be useful to my understanding.  As we go through the interview, there may be questions that you would rather not answer. In this case, please feel free to "pass" on that question. Also, if any question is unclear, don’t be afraid of telling me so. I will do my best to ask it more clearly.  Please be assured that your responses and all the information/data you provide are completely confidential and anonymous. Your participation in this research study is voluntary. You may withdraw at any time and for any reason without an explanation.  While listening I might be taking some notes to ask you some follow-up questions.  However, to be fully engaged in the interview and for research purposes, I would like to audio-record our conversation. Is that alright with you?  Then just one last reminder: there are no right or wrong answers; I am only interested in listening to what you have to say. | |
| --- | --- | --- |
|  | **Questions and follow-up questions** | Comment to the interviewer |
| Ice braking | Before we begin, I would like to get to know you a little better. | |
|  | What was happen to be admitted in the hospital? | |
| After discharge | What was it like for you during the first few days at home after leaving the hospital?  What helped you most once you returned home, and what was difficult? |  |
| Receive and understand information | In the hospital, did you fully understand what was happening? And what will happen after discharge? did any healthcare professionals explain what you might needed to do to take care of yourself at home? How?  In the hospital, do you think the healthcare professionals explain things in a way you could understand?  **Before you left the hospital, did you get information about**   - what symptoms to look out for at home? - prescription or over-the-counter medicine you should take at home? - the possible side effects of each medicine clear to you? - received written information about how to take care of yourself at home - the things that can make condition get better or worse |  |
| Self- management | Did you have been told results of tests, changes in medication, arranged for future tests/monitoring of condition?  Are you confidence in your ability to manage medications, such as using, remembering to take them all and take them at the right time?  What was the support you received from the doctor to help your selfcare?  What kind of support you need to facilitate your care for yourself? | Self-management is a process through which individuals actively cope with their chronic disease in the context of their daily lives |
| Caregiver involvement | Did they involve a family member or other caregiver in planning care after discharge? How?  Matching of discharge care plan to the level of support from family, friends, and others during recovery |  |
| Relationship with the healthcare providers | How did the way staff treated you make you feel?  In the hospital, was there anything that you would have liked done better or differently?  In your mind, what do people care most about and with about health care provider? |  |
| Relationship with primary healthcare service | In the service, what did you liked and dislike about the hospital  In your mind, what do people care most about and wish from health service to control their disease? |  |
| Cost of treatment | Do you have health insurance?  Does it cover your all medications and investigations?  Type of insurance  What kind of the services you would pay for? |  |
| Before finalization | Age and gender |  |
|  | Education level |  |
|  | Marital status |  |
|  | Type of illness |  |
|  | **Self-reported health**   - In the past week, how would you rate your physical health? - emotional health, including your mood and your ability to think? - In the past week, how would you rate your sleep? - In the past week, how often have you had bodily pain? - In the past week, to what extent were you able to carry out your everyday physical activities such as walking, climbing stairs, carrying groceries, or moving a chair?   In the past week, how would you rate your physical health? |  |
|  | How confident are you in filling out medical forms by yourself?  (information about health literacy) |  |
|  | Health insurance: Yes, No |  |
| Finalization | **What could have made your transitional care experience better?**  **Is there anything that I have not asked about your experience and the support you have found or would like to find, that you would like to share?**  I would like to finish this interview by thanking you for participating in this research. |  |
