# Supplementary material for: The Needs of Patients With Chronic Disease in Transitional Care From Hospital to Home in Sudan: A Qualitative Study
Source: Health Serv Insights. 2024 May 14;17:11786329241249282. doi: 10.1177/11786329241249282 (PMC11092543; doi:10.1177/11786329241249282)
Supplement: sj-docx-3-his-10.1177_11786329241249282 – Supplemental material for The Needs of Patients With Chronic Disease in Transitional Care From Hospital to Home in Sudan: A Qualitative Study [file sj-docx-3-his-10.1177_11786329241249282.docx]

**Supp 3.** Characteristics of the study participants

|  | Age | Gender | Marital Status | Type of Chronic Disease | Employment | Education Level | Financial Supporter | Hospital of Admission | Type of The Hospital |
| --- | --- | --- | --- | --- | --- | --- | --- | --- | --- |
| *P1* | 70 | Female | Married | CKD/ DM type 2 | Housewife | Primary School | Self /Sister | Bahri Hospital | General Public Hospital |
| *P2* | 67 | Male | Married | RCC | Not Employed | Non-Formal Education (Khlalwa) | Son | Bahri Hospital | General Public Hospital |
| *P3* | 65 | Female | Married | DM type 2/ HTN | Housewife | Not Educated | Family | Bahri Hospital | General Public Hospital |
| *P4* | 23 | Female | Single | DM type 2 | Student | University Graduate | Mother | Almualim Hospital | Private Hospital |
| *P5* | 56 | Female | Married | DM type 2 | Housewife | Not Educated | Son/ Husband | Ibrahim Malik Hospital | General Public Hospital |
| *P6* | 38 | Male | Single | Liver failure | Worker | Secondary School | Mother/ Self | Ibrahim Malik Hospital | General Public Hospital |
| *P7* | 38 | Female | Married | CCD/ DM type 2 | Housewife | Primary School | Family | Ahmed Qasim Cardiac Center | Specialized Public Hospital |
| *P8* | 54 | Male | Married | HTN | Mosque Emam | Non-Formal Education (Khlalwa) | Self | Ibrahim Malik Hospital | General Public Hospital |
| *P9* | 55 | Female | Married | CCD/ HTN | Housewife | Secondary School | Husband | Ibrahim Malik Hospital | General Public Hospital |
| *P10* | 65 | Male | Married | IHD | Married | - | Son | [Royal Care International Hospital](https://www.facebook.com/royalcare.sd/) | Private Hospital |
| *P11* | 51 | Female | Married | IHD | Teacher | University Graduate | Family/ Self | Sudan Heart Center | Specialized Military Hospital |
| *P12* | 66 | Female | Married | DM type 2 | Retired | Post University Graduate | Family/ Self | Almualim Hospital | Private Hospital |
| *P13* | 37 | Female | Single | CHF | Not Employed | Secondary School | Family | Ahmed Qasim Cardiac Center | Specialized Public Hospital |
| *P14* | 45 | Female | Married | CHF | Housewife | Not Educated | Family | Ahmed Qasim Cardiac Center | Specialized Public Hospital |
| *P15* | – | Male | Married | CHF | Manager | University Graduate | Son/ Self | Ahmed Qasim Cardiac Center | Specialized Public Hospital |
| *P16* | 63 | Male | Married | CKD | Not Employed | Primary School | Family | Bahri Hospital | General Public Hospital |
| *P17* | 23 | Female | Single | DM type 1 | Student | University Graduate | Family | Almualim Hospital | Private Hospital |

CKD=Chronic Kidney Disease, DM=Diabetes Mellitus, RCC=Renal Cell Carcinoma, CCD=Chronic Cardiac Disease, HTN=Hypertension, IHD, Ischemic Heart Disease, CHF=Congestive Heart Failure
